# Experience with ocular surface disease index analysis to assess dry eye disease and related conditions in Lagos, Nigeria

**DOI:** 10.11604/pamj.2026.53.169.49506

**Published:** 2026-04-21

**Authors:** Olubanke Theodora Ilo, Olufemi Emmanuel Babalola, Uyiekpen Ima-Edonwonyi, Olusegun Jacobs Abayomi, Abdul-Warith Olaitan Akinshipo, Gbenga Jonathan Babalola

**Affiliations:** 1 Guinness Eye Centre, Lagos University Teaching Hospital, Lagos, Nigeria,; 2 Department of Surgery, Bingham University and Rachel Eye Centre, Abuja, Nigeria,; 3 Department of Rheumatology, Lagos University Teaching Hospital, Lagos, Nigeria,; 4 Department of Dentistry, Lagos University Teaching Hospital, Lagos, Nigeria,; 5 College of Medicine, University of Lagos, Lagos, Nigeria

**Keywords:** Dry eye disease, ocular surface disease index, Schirmer test, Sjögren’s syndrome, tear break-up time

## Abstract

**Introduction:**

dry eye disease (DED) is a multifactorial ocular surface disorder that remains underdiagnosed, particularly in sub-Saharan Africa, where epidemiological data are limited. This study aimed to describe the distribution and severity of DED using the Ocular Surface Disease Index (OSDI), and to evaluate its association with demographic factors and objective diagnostic tests among adults in Lagos, Nigeria.

**Methods:**

we conducted a hospital-based cross-sectional study involving 173 consecutive participants recruited from ophthalmology, rheumatology, and dental clinics in Lagos, Nigeria. Participants completed the OSDI questionnaire and underwent Schirmer´s test and Tear Break-Up Time (TBUT) assessment. Demographic and clinical variables were analysed using STATA version 16.1. The sensitivity, specificity, and predictive values of OSDI were evaluated against objective measures of ocular dryness.

**Results:**

the mean OSDI score was 21.2 ± 18.9. Forty percent of participants had scores within the normal range, while 24.3% had severe DED. Higher OSDI scores were observed among females, older participants, and those with a prior diagnosis of dry eye or systemic dryness symptoms, although not all associations reached statistical significance. When compared with objective tests, OSDI demonstrated high sensitivity but low specificity (7-13%).

**Conclusion:**

dry eye disease is common among adults attending tertiary clinics in Lagos and presents with heterogeneous ocular and systemic features. While OSDI is a useful high-sensitivity screening tool, its low specificity underscores the need for confirmatory objective testing. Larger population-based studies are warranted to better define the epidemiology and determinants of DED in Nigeria.

## Introduction

Dry eye disease is a common, chronic, and multifactorial disorder of the ocular surface that may significantly impair visual function and quality of life. Hospital-based studies from Nigeria have demonstrated that DED is prevalent and frequently underdiagnosed in clinical practice [1]. Similar findings have been reported in other African and low- and middle-income settings, highlighting the growing public health relevance of the condition [2]. To improve the assessment of dry eye symptoms and their functional impact, several symptom-based questionnaires have been developed. One of the most widely used is the OSDI, developed by the Outcomes Research Group at Allergan Inc., which provides a rapid and reproducible measure of ocular symptoms, vision-related function, and environmental triggers [3]. The reliability and validity of the OSDI have been well established, making it a useful tool in both clinical and research settings. Dry eye disease is currently defined by the Tear Film and Ocular Surface Society (TFOS) as a multifactorial disease of the ocular surface characterised by loss of tear film homeostasis, tear film instability and hyperosmolarity, ocular surface inflammation and damage, and neurosensory abnormalities [4]. This complex pathophysiology contributes to the frequently observed discordance between patient-reported symptoms and objective clinical signs. Other instruments have been developed to assess patients' subjective experience of visual function and ocular discomfort, including the National Eye Institute Visual Function Questionnaire-25 (NEI VFQ-25), which evaluates vision-related quality of life [5]. However, due to its brevity, multidimensional structure, and ease of administration, the OSDI has gained wider acceptance for routine dry eye assessment.

Epidemiological studies from different regions have demonstrated substantial variability in the prevalence and severity of DED. Large population-based studies among women in the United States have shown a high burden of dry eye symptoms [6]. Similar associations between dry eye symptoms and impaired vision-related quality of life have been reported in other populations [7]. Occupational exposure, particularly among visual display terminal users, has also been identified as an important risk factor for DED [8]. Hospital-based studies from India have further highlighted the burden of dry eye disease in clinical populations [9]. Iatrogenic causes and treatment-related ocular surface changes have also been recognised as contributors to DED [10]. In the Middle East, population-based studies have identified both environmental and demographic risk factors influencing dry eye symptoms [11]. Early clinical trial frameworks and definitions for dry eye disease emphasised the importance of combining symptom assessment with objective testing [12]. Subsequent validation studies confirmed the repeatability and responsiveness of the OSDI in different clinical contexts [13]. Consensus reports from the Asia Dry Eye Society further refined diagnostic approaches and highlighted regional variations in disease presentation [14]. The relationship between dry eye symptoms and ageing remains complex. Some studies have reported a reduction in symptom reporting with increasing age, suggesting altered symptom perception or adaptation [15]. In contrast, studies among visual display terminal users and general populations have reported increasing prevalence of DED with age [16,17]. Population-based studies in elderly cohorts have also demonstrated a high burden of dry eye disease among older adults [18].

Additional epidemiological studies have shown that dry eye symptoms are common in older populations and may be influenced by systemic health and environmental factors [19]. Age-related changes in tear composition and protein profiles have also been implicated in symptom variability [20]. Sex-related differences in dry eye disease have been widely reported. Large epidemiological studies have consistently shown a higher prevalence of dry eye symptoms among women [21]. Similar findings have been observed in population-based studies examining risk factors for dry eye syndrome [22]. However, some studies from Southeast Asia have reported weaker or inconsistent sex-related differences [23]. Variability in diagnostic criteria and study design may partly explain these discrepancies [24].

Systemic conditions such as hypertension have been associated with increased severity of dry eye disease in large population-based studies [25]. Diabetes mellitus has also been linked to a higher prevalence of dry eye symptoms and ocular surface abnormalities [26]. Clinical reviews have highlighted the importance of recognising systemic comorbidities in the diagnosis and management of dry eye disease [24]. Hormonal factors, particularly menopause, have been implicated in dry eye pathophysiology. Epidemiological studies have demonstrated an increased prevalence of dry eye syndrome among postmenopausal women [27]. However, interventional studies evaluating hormone replacement therapy have yielded inconsistent results, suggesting a complex and multifactorial relationship [25].

Objective diagnostic tests for dry eye disease, including Schirmer's test and TBUT, show considerable variability over time, underscoring the challenges of relying on single measurements [25]. In autoimmune conditions such as Sjögren´s syndrome, patients consistently demonstrate higher OSDI scores and more severe ocular and systemic dryness [28].

Beyond ocular symptoms, dry eye disease is increasingly recognised as part of a broader spectrum of systemic dryness. Studies have demonstrated associations between DED and systemic comorbidities affecting other mucosal surfaces [27]. Long-term clinical studies of Sjögren´s syndrome further highlight the multisystem nature of the disease [29]. Non-ocular dryness, including oral and skin dryness, has been reported in patients with dry eye disease even in the absence of overt autoimmune disease [29]. Strong correlations between ocular and systemic dryness have been documented in Sjögren´s syndrome and related conditions [30]. In Nigeria, data on the performance of symptom-based screening tools such as the OSDI in relation to objective diagnostic tests remain limited. This study, therefore, reports our experience using the Ocular Surface Disease Index among adults attending tertiary healthcare clinics in Lagos, Nigeria. Conducted within a broader research framework on Sjögren´s syndrome, the present manuscript focuses specifically on the distribution and severity of dry eye symptoms measured by OSDI, their association with demographic and clinical factors, and the screening performance of OSDI in comparison with commonly used objective diagnostic tests.

## Methods

**Study design and setting:** this was a hospital-based cross-sectional study conducted in Lagos, Nigeria. Participants were recruited from the ophthalmology clinic of Guinness Eye Hospital, Lagos University Teaching Hospital, as well as from affiliated rheumatology and dental clinics. The study was conducted between May 2023 and October 2023. The study formed part of a broader research programme investigating Sjögren´s syndrome and related dryness disorders; however, the present analysis focused specifically on the assessment of dry eye symptoms using the OSDI and their relationship with demographic factors and objective measures of ocular and systemic dryness.

**Study population and sampling strategy:** a total of 173 consecutive participants were recruited using a non-probability consecutive sampling method. Participants were enrolled from three clinical services: ophthalmology (n = 157), rheumatology (n = 9), and dental/oral medicine clinics (n = 7). This recruitment strategy was intentionally adopted to capture a broad spectrum of ocular and systemic dryness presentations, including individuals with symptoms suggestive of autoimmune disease. Because recruitment was hospital-based, the study population was not intended to be representative of the general Lagos population. The potential for selection bias is acknowledged, and findings are interpreted within this context.

### Eligibility criteria

**Inclusion criteria:** nigerian residents aged 13 years and above, residing in Lagos for at least one year. Willingness to provide written informed consent (or assent with parental consent for minors).

**Exclusion criteria:** active ocular infection at the time of examination; ocular surgery within the preceding six months; use of topical ocular medications or systemic drugs known to significantly affect tear production within the preceding three months; although dry eye disease is uncommon in adolescents, individuals aged 13-17 years were included to avoid arbitrary age exclusion and because they constituted a very small proportion of the sample. Age-stratified analyses were performed to minimise distortion of results.

**Sample size considerations:** the sample size of 173 participants was considered adequate for an exploratory cross-sectional analysis examining associations between OSDI scores and multiple demographic and clinical variables. This sample size is consistent with similar hospital-based studies evaluating dry eye disease and symptom-based questionnaires in comparable settings [1,2,9].

**Data collection procedures:** demographic and clinical data: participants provided demographic information, including age, sex, and occupation. Clinical history included symptoms of ocular dryness, prior diagnosis of dry eye disease, systemic symptoms of dryness (oral, throat, vaginal, or skin dryness), history of autoimmune disease, diabetes mellitus, hypertension, alcohol use, and visual display unit exposure.

**Ocular Surface Disease Index (OSDI):** all participants completed the OSDI questionnaire. The OSDI consists of 12 items assessing ocular symptoms, vision-related function, and environmental triggers. Responses are scored on a Likert scale ranging from 0 (none of the time) to 4 (all of the time).

The OSDI score was calculated using the standard formula: OSDI = (sum of scores × 25) / number of questions answered. Scores were categorised as follows: 0-12: Normal; 13-22: Mild dry eye; 23-32: Moderate dry eye; 33-100: Severe dry eye. Where necessary, the questionnaire was translated into the local language (Yoruba) with assistance from trained staff to ensure comprehension.

**Objective clinical assessments:** objective tests of ocular dryness included Schirmer's test and TBUT, performed according to standard clinical protocols. Where applicable, measures of salivary gland function (stimulated and unstimulated sialometry) and systemic disease activity scores (ESSDAI) were obtained as part of the broader Sjögren´s syndrome evaluation.

**Statistical analysis:** data were analysed using Stata version 16.1 (StataCorp, Texas, USA). Continuous variables were summarised using means and standard deviations, while categorical variables were summarised using frequencies and percentages. Internal consistency of the OSDI questionnaire was assessed using Cronbach´s alpha. Associations between OSDI scores and continuous variables were explored using correlation analysis. Comparisons of mean OSDI scores across demographic and clinical groups were performed using t-tests or analysis of variance (ANOVA) as appropriate. For categorical analyses, OSDI scores were dichotomised as normal versus abnormal. Chi-square tests were used to assess associations between OSDI performance and clinical variables. Binary logistic regression was employed to estimate odds ratios and 95% confidence intervals for factors associated with abnormal OSDI scores. The sensitivity, specificity, positive predictive value, and negative predictive value of OSDI were calculated using objective clinical tests as reference standards. Statistical significance was defined as a p-value <0.05. Findings with p-values above this threshold were interpreted cautiously and described as trends.

**Ethical considerations:** the study was conducted in accordance with the Declaration of Helsinki and was approved by the Ethical Review Board of Lagos University Teaching Hospital. Written informed consent was obtained from all participants before enrolment. Participants were assured of confidentiality and informed of their right to withdraw from the study at any time without prejudice to their care.

## Results

**Participant characteristics:** a total of 173 participants were enrolled in the study. Of these, 71 (41.0%) were male, and 102 (59.0%) were female. The mean age of participants was 48.3 years (SD 17.3; 95% CI 45.7-50.9), with an age range of 13 to 86 years. Most participants were recruited from the ophthalmology clinic (n = 157), while a smaller proportion were enrolled from the rheumatology (n = 9) and dental/oral medicine (n = 7) clinics.

**Feasibility and completion of the OSDI questionnaire:** all participants successfully completed the OSDI questionnaire. The average completion time was approximately 20-25 minutes. Assistance with translation into the Yoruba language was required in approximately 5% of cases, primarily among older participants.

**Figure 1:**
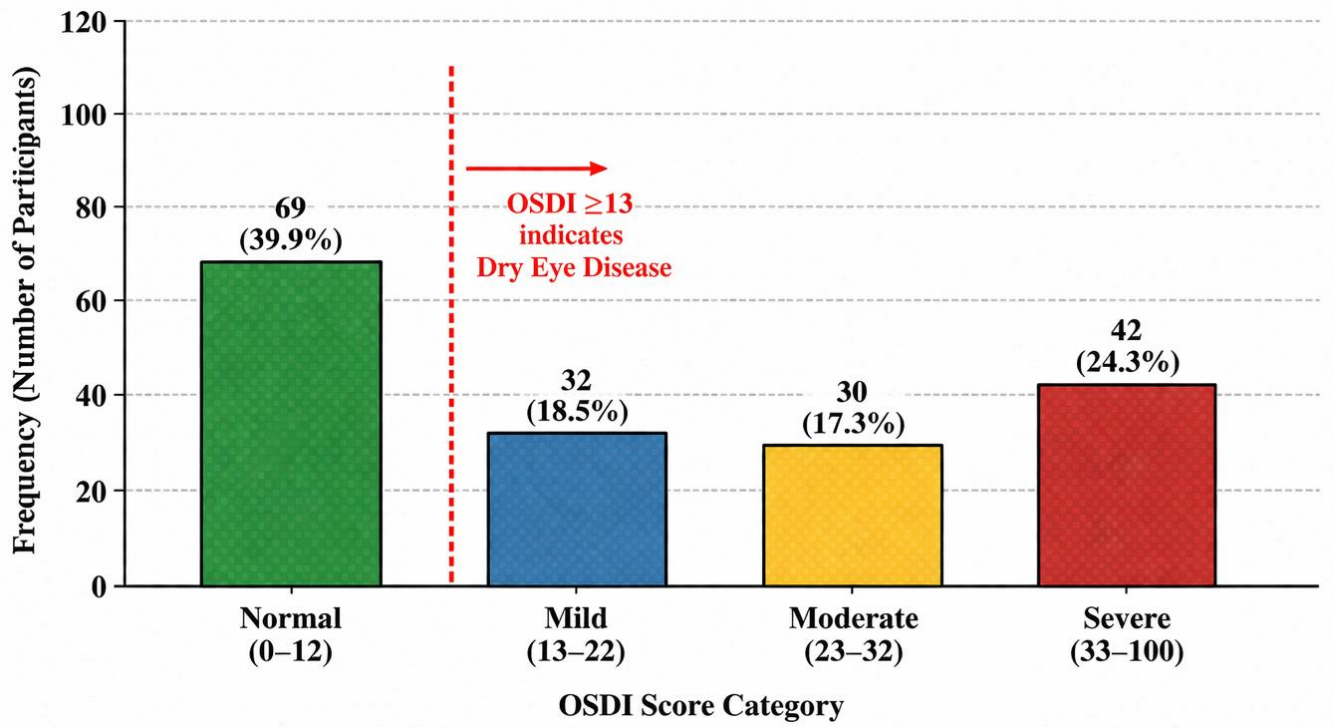
distribution of ocular surface disease index scores in the study population in Lagos State

**Distribution of OSDI scores:** the mean OSDI score for the study population was 21.2 ± 18.9 (95% CI 18.4-24.1), with scores ranging from 0 to 87.5. Based on standard OSDI categories, 40.0% of participants had scores within the normal range, 11.6% had mild dry eye, 24.3% had moderate dry eye, and 24.3% had severe dry eye. The distribution of OSDI scores is summarised in Table 1 and illustrated in Figure 1.

**Table 1:** performance of 173 respondents on the ocular surface disease index test in Lagos

Dry eye category by OSDI score	Range	Number of patients within range	Percentage
Normal	12 or less	69	39.9
Mild	13-22	42	24.3
Moderate	23-32	20	11.6
Severe	33-100	42	24.3
**Total**		**173**	**100**

**Comparison of OSDI scores with other populations:** when compared with published data from other countries, the mean OSDI score in this Lagos cohort was lower than those reported in several international studies. The closest comparable mean score was observed in studies from China, with no statistically significant difference (p = 0.098), while the mean score differed significantly from that reported in India (p < 0.0001). A higher proportion of participants in this study fell within the normal OSDI category, and a lower proportion had severe disease compared with most comparator populations (Table 2).

**Table 2:** comparison of ocular surface disease index scores in various countries

Population	Study sample size	Average OSDI score (mean ± SD)	Severity distribution (% of total)	Reference
**Lagos, Nigeria**	173	21.2 ± 18.9	Normal: 39.9%, Mild: 24.3%, Moderate: 11.6%, Severe: 24.3%	Current Study
**United States**	300	28.7 ± 16.1	Normal: 8%, Mild: 20%, Moderate: 32%, Severe: 40%	Lemp [6]
**China**	450	23.5 ± 13.2	Normal: 10%, Mild: 25%, Moderate: 40%, Severe: 25%	Tsubota *et al*. [7]
**Japan**	200	35.2 ± 11.7	Normal: 5%, Mild: 15%, Moderate: 30%, Severe: 50%	Seth *et al*. [8]
**India**	250	27.8 ± 14.9	Normal: 7%, Mild: 18%, Moderate: 35%, Severe: 40%	Han *et al*. [9]
**Brazil**	220	30.1 ± 15.3	Normal: 6%, Mild: 22%, Moderate: 32%, Severe: 40%	Chia *et al*. [10]
**Saudi Arabia**	180	29.3 ± 14.1	Normal: 9%, Mild: 21%, Moderate: 35%, Severe: 35%	Zhou *et al*. [11]

**Figure 2:**
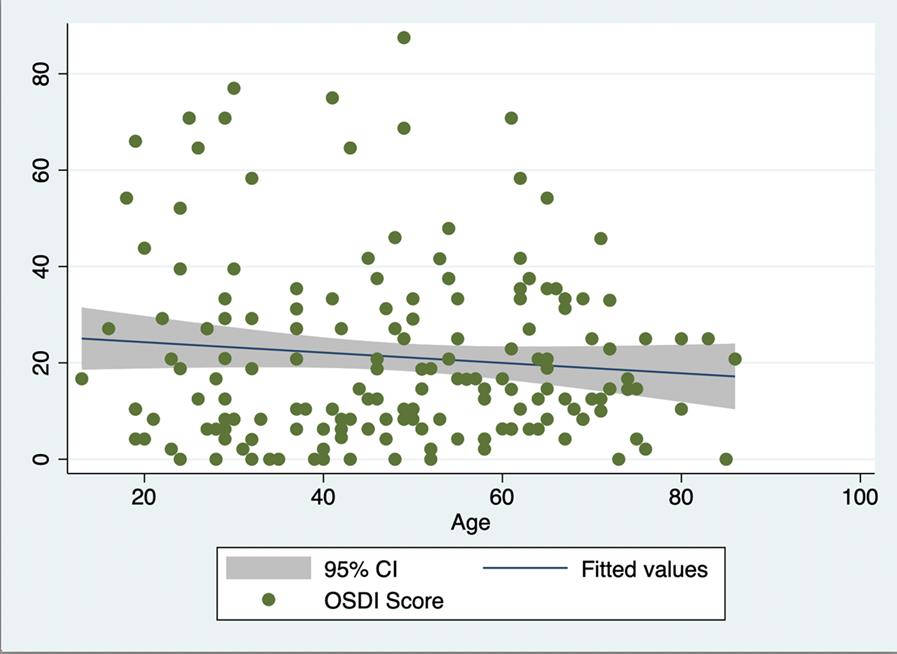
regression of ocular surface disease index score against age

**Relationship between OSDI scores and age:** regression analysis of OSDI score against age as a continuous variable demonstrated a weak negative correlation (Bonferroni-adjusted correlation coefficient = -0.0985; p = 0.1974), indicating that increasing age was not significantly associated with higher symptom severity (Figure 2). However, when age was analysed categorically, 65.1% of participants aged >44 years had abnormal OSDI scores compared with 52.7% of those aged ≤44 years. This difference did not reach statistical significance (X^2^ = 2.58; p = 0.108). In binary logistic regression, participants in the older age group were 66% more likely to have an abnormal OSDI score (OR 1.66; 95% CI 0.89-3.11; p = 0.109), though this association was not statistically significant (Table 3).

**Table 3:** predictive value of ocular surface disease index score for some objective parameters

Objective parameter	Positive predictive value %	Negative predictive value %	Sensitivity %	Specificity %
Subnormal stimulated sialometry	57.1	50.0	94.1	7.7
Subnormal unstimulated sialometry	90.9	5.0	34.5	50.0
Presence of corneal staining	19.1	88.0	47.1	66.0
Subnormal Schirmer test RE	25.0	92.3	96.3	13.3
Subnormal tear breakup-time RE	18.3	100	100	13.3
Subnormal ESSDAI score	15.4	98.6	94.1	43.6

**Sex and OSDI performance:** the mean OSDI score was higher among females compared with males (22.6 vs 19.3), but this difference was not statistically significant (p = 0.255). Females were more likely to have abnormal OSDI scores than males (OR 1.59; 95% CI 0.85-2.98), although this association did not reach statistical significance (p = 0.140).

**Clinical and systemic factors associated with OSDI scores:** the relationship between selected demographic and clinical variables and mean OSDI scores. Diabetic status, hypertensive status, age group, sex, and alcohol use were not significantly associated with the mean OSDI score. Menopausal women had lower mean OSDI scores compared with non-menopausal women, and this difference reached statistical significance. Participants with a prior diagnosis of dry eye disease had significantly higher mean OSDI scores compared with those without such a history. Participants with subnormal TBUT, a prior diagnosis of xerostomia, or a prior diagnosis of dry eye tended to have higher OSDI scores; however, not all of these differences reached statistical significance.

**Associations between OSDI performance and clinical diagnoses:** associations between abnormal OSDI scores and selected clinical variables. Abnormal OSDI performance was significantly associated with: prior physician diagnosis of dry eye disease; self-reported history of dry eye symptoms; diagnosis of Sjögren´s syndrome; history of oral dryness; abnormal ESSDAI score. Participants with abnormal OSDI scores were nearly five times more likely to have a prior diagnosis of dry eye disease and approximately four times more likely to have a subnormal Schirmer test. Although diabetics and hypertensive participants were more likely to have abnormal OSDI scores, these associations did not reach statistical significance.

**Predictive performance of OSDI:** the screening performance of OSDI against objective measures of dryness. OSDI demonstrated high sensitivity for subnormal unstimulated sialometry (91.0%), stimulated sialometry (94.1%), subnormal Schirmer test (96.3%), subnormal TBUT (100%), and abnormal ESSDAI score (94.1%). However, OSDI demonstrated low specificity, ranging from 7.7% to 13.3%, across several objective parameters. Negative predictive values were high for corneal staining, subnormal Schirmer test in the right eye, subnormal TBUT in the right eye, and abnormal ESSDAI score.

**Systemic dryness and OSDI performance:** participants reported dryness affecting the mouth, throat, vagina, and skin, either singly or in combination. The proportion of participants with abnormal OSDI scores increased progressively with the number of reported dry areas. This trend was statistically significant (Pearson's X^2^ = 23.5; p < 0.0001).

## Discussion

This study provides hospital-based data on the distribution, severity, and clinical correlates of DED among adults attending tertiary healthcare clinics in Lagos, Nigeria, using the OSDI alongside objective diagnostic tests. In a setting where epidemiological data on DED remain limited [1,2], these findings contribute important contextual evidence on the utility and limitations of symptom-based screening tools in an African population.

**Distribution and severity of dry eye symptoms:** the mean OSDI score observed in this cohort indicates a substantial burden of dry eye symptoms, with approximately one quarter of participants classified as having severe disease. Although a sizeable proportion of participants fell within the normal OSDI range, the overall distribution underscores the heterogeneity of symptom severity among clinic attendees. Similar variability has been reported in hospital-based and population-based studies from other regions [6-11], reflecting the multifactorial nature of DED and differences in environmental exposure, health-seeking behaviour, and comorbid conditions.

When compared with published international data, the mean OSDI score in this Lagos cohort was lower than those reported in several other populations, and most closely approximated values reported from China [7,14]. These differences should be interpreted cautiously, as climatic conditions, occupational exposure, diagnostic thresholds, and cultural differences in symptom perception may all influence reported symptom severity.

**Age and sex-related findings:** the relationship between age and dry eye symptoms in this study was complex. Regression analysis suggested a weak inverse relationship between age and OSDI score, whereas categorical analyses demonstrated a higher prevalence of abnormal OSDI scores among older participants. Similar inconsistencies have been reported in the literature, with some studies demonstrating increasing prevalence of DED with age [16-19], while others suggest reduced symptom reporting in older individuals, possibly due to altered symptom perception or adaptation [15,20]. These findings highlight the importance of distinguishing between symptom intensity and disease prevalence when interpreting age-related patterns. Female participants had higher mean OSDI scores and were more likely to have abnormal OSDI performance than males, although these differences did not reach statistical significance. This trend is consistent with numerous epidemiological studies reporting a higher burden of dry eye symptoms among women [6,21,22]. However, other studies have shown weaker or inconsistent sex differences [23,24], suggesting that hormonal, environmental, and behavioural factors may interact in complex ways.

**Menopausal status:** an unexpected finding in this study was the observation of lower mean OSDI scores among menopausal women compared with non-menopausal women. This contrasts with several epidemiological studies that have reported an increased prevalence of dry eye symptoms after menopause [20]. However, interventional studies examining hormone replacement therapy have produced inconsistent results [20], suggesting that the relationship between menopausal status and dry eye symptoms may be more nuanced. Possible explanations for the present finding include differences in symptom perception, health-seeking behaviour, or unmeasured confounding factors. This observation should therefore be regarded as hypothesis-generating rather than definitive.

**Systemic conditions and ocular surface disease:** although diabetes mellitus and hypertension were associated with higher odds of abnormal OSDI scores, these associations did not reach statistical significance. Prior studies have reported variable associations between systemic conditions and dry eye disease [25-27], underscoring the heterogeneity of study populations and diagnostic approaches. Nevertheless, the observed trends support the importance of considering systemic health in the evaluation of patients with dry eye symptoms.

**OSDI and objective diagnostic tests:** a key finding of this study was the high sensitivity but very low specificity of OSDI when compared with objective diagnostic tests such as Schirmer´s test and Tear Break-Up Time. This finding is consistent with prior reports demonstrating poor concordance between subjective symptoms and objective signs in DED [12,20]. While the high sensitivity of OSDI supports its role as a screening tool, the low specificity observed in this study reinforces that OSDI should not be used as a standalone diagnostic test. Objective assessments remain essential for accurate diagnosis and disease classification.

**Systemic dryness and autoimmune disease:** the progressive increase in abnormal OSDI performance with an increasing number of reported dry areas highlights the multisystem nature of dryness disorders. Similar associations between ocular and non-ocular dryness have been described in patients with Sjögren´s syndrome and related conditions [21-30]. The strong relationship between OSDI scores and systemic dryness symptoms in this cohort underscores the importance of considering broader mucosal involvement when evaluating patients presenting with dry eye symptoms.

**Strengths and limitations:** the strengths of this study include the use of a validated symptom questionnaire alongside multiple objective diagnostic measures and the inclusion of participants from different clinical specialities to capture a broad spectrum of dryness-related conditions. However, several limitations should be acknowledged. The hospital-based design limits generalisability to the wider population, and the cross-sectional nature of the study precludes causal inference. Additionally, advanced diagnostic modalities such as tear osmolarity testing or meibography were not available. Despite these limitations, the study provides valuable baseline data from a region where evidence remains scarce.

**Implications for clinical practice and research:** the findings of this study support the use of OSDI as a practical, high-sensitivity screening tool for dry eye disease in resource-limited settings, while emphasising the necessity of confirmatory objective testing. Future population-based studies are needed to better define the epidemiology of DED in Nigeria and to explore the interplay between ocular symptoms, systemic disease, and environmental exposures.

## Conclusion

This hospital-based cross-sectional study demonstrates that dry eye disease is common among adults attending tertiary healthcare clinics in Lagos, Nigeria, with a wide spectrum of symptom severity and associated ocular and systemic features. The Ocular Surface Disease Index proved to be a practical and highly sensitive tool for identifying individuals with dry eye symptoms in this setting; however, its low specificity highlights the necessity of confirmatory objective testing for accurate diagnosis and classification. Associations between abnormal OSDI scores and demographic factors, prior dry eye diagnosis, systemic dryness symptoms, and autoimmune disease underscore the multifactorial and multisystem nature of dry eye disease. The observed relationships between ocular symptoms and non-ocular dryness further emphasise the importance of considering broader systemic involvement, particularly in patients with suspected Sjögren´s syndrome. These findings contribute valuable baseline data from a region where epidemiological evidence on dry eye disease remains limited. Larger, population-based studies incorporating comprehensive diagnostic modalities are warranted to better define the burden, determinants, and clinical implications of dry eye disease in Nigeria and similar settings.

### What is known about this topic

- Dry eye disease is a multifactorial ocular surface disorder that is often underdiagnosed;
- The OSDI is a widely used questionnaire for evaluating the severity of DED symptoms;
- Objective tests such as Schirmer´s and TBUT are essential for confirming diagnosis.

### What this study adds

- This study provides the first data on the prevalence and clinical features of DED among Nigerian adults using both OSDI and objective tests;
- It demonstrates significant associations between DED severity and demographic factors such as age, sex, and menopausal status;
- Furthermore, it highlights the value and limitations of OSDI as a screening tool in an African population context.

## References

[1] Onwubiko SN, Eze BI, Udeh NN, Arinze OC, Onwasigwe EN, Umeh RE (2014). Dry eye disease: prevalence, distribution and determinants in a hospital-based population. Cont Lens Anterior Eye.

[2] Echieh CI, Etim BA, Echieh CP, Oyeniyi T, Ajewole J (2021). A comparative assessment of dry eye disease among outdoor street sweepers and indoor office cleaners. BMC Ophthalmol.

[3] Schiffman RM, Christianson MD, Jacobsen G, Hirsch JD, Reis BL (2000). Reliability and validity of the Ocular Surface Disease Index. Arch Ophthalmol.

[4] Craig JP, Nichols KK, Akpek EK, Caffery B, Dua HS, Joo CK (2017). TFOS DEWS II Definition and Classification Report. Ocul Surf.

[5] Mangione CM, Lee PP, Gutierrez PR, Spritzer K, Berry S, Hays RD (2001). National Eye Institute Visual Function Questionnaire Field Test Investigators. Development of the 25-item National Eye Institute Visual Function Questionnaire. Arch Ophthalmol.

[6] Lemp MA (1995). Report of the National Eye Institute/Industry workshop on Clinical Trials in Dry Eyes. CLAO J.

[7] Tsubota K, Yokoi N, Shimazaki J, Watanabe H, Dogru M, Yamada M (2017). New Perspectives on Dry Eye Definition and Diagnosis: A Consensus Report by the Asia Dry Eye Society. Ocul Surf.

[8] Seth M, Michael W, Divya N, George W (2021). Relationship between dry eye symptoms and ageing. Investigative Ophthalmology & Visual Science.

[9] Han SB, Hyon JY, Woo SJ, Lee JJ, Kim TH, Kim KW (2011). Prevalence of dry eye disease in an elderly Korean population. Arch Ophthalmol.

[10] Chia EM, Mitchell P, Rochtchina E, Lee AJ, Maroun R, Wang JJ (2003). Prevalence and associations of dry eye syndrome in an older population: the Blue Mountains Eye Study. Clin Exp Ophthalmol.

[11] Zhou L, Beuerman RW, Chan CM, Li SF, Zhu G, Zhao SZ (2016). Age-related changes in the tear protein profile and their relation to OSDI. Ophthalmology.

[12] Moss SE, Klein R, Klein BE (2000). Prevalence of and risk factors for dry eye syndrome. Arch Ophthalmol.

[13] Lee AJ, Lee J, Saw SM, Gazzard G, Koh D, Widjaja D (2002). Prevalence and risk factors associated with dry eye symptoms: a population based study in Indonesia. Br J Ophthalmol.

[14] Gayton JL, Shoja MR, Ali TK (2013). Dry eye syndrome: pathophysiology, classification, and diagnosis. American Journal of Ophthalmology.

[15] Ayaki M, Kawashima M, Negishi K, Kishimoto T, Mimura M, Tsubota K (2016). Sleep and mood disorders in dry eye disease and allied irritating ocular diseases. Sci Rep.

[16] Manaviat MR, Rashidi M, Afkhami-Ardekani M, Shoja MR (2008). Prevalence of dry eye syndrome and diabetic retinopathy in type 2 diabetic patients. BMC Ophthalmol.

[17] Kaštelan S, Tomić M, Salopek-Rabatić J, Novak B (2013). Diagnostic procedures and management of dry eye. Biomed Res Int.

[18] Schaumberg DA, Sullivan DA, Dana MR (2002). Epidemiology of dry eye syndrome. Adv Exp Med Biol.

[19] Schaumberg DA, Buring JE, Sullivan DA, Dana MR (2001). Hormone replacement therapy and dry eye syndrome. JAMA.

[20] Sullivan BD, Crews LA, Sonmez B, de la Paz MF, Comert E, Charoenrook V (2012). Clinical utility of objective tests for dry eye disease: variability over time and implications for clinical trials and disease management. Cornea.

[21] Kalk WW, Mansour K, Vissink A, Spijkervet FK, Bootsma H, Kallenberg CG (2002). Oral and ocular manifestations in Sjögren's syndrome. J Rheumatol.

[22] Kawashima M, Yamada M, Shigeyasu C, Suwaki K, Uchino M, Hiratsuka Y (2020). Association of Systemic Comorbidities with Dry Eye Disease. J Clin Med.

[23] Vitali C, Bombardieri S, Jonsson R, Moutsopoulos HM, Alexander EL, Carsons SE (2002). European Study Group on Classification Criteria for Sjögren´s syndrome: classification criteria and long-term clinical studies. Ann Rheum Dis.

[24] Shah S, Jani H (2015). Prevalence and associated factors of dry eye: Our experience in patients above 40 years of age at a Tertiary Care Center. Oman J Ophthalmol.

[25] Akpek EK, Mathews P, Hahn S, Hessen M, Kim J, Grader-Beck T (2015). Ocular and systemic morbidity in a longitudinal cohort of Sjögren's syndrome. Ophthalmology.

[26] Uchino M, Schaumberg DA, Dogru M, Uchino Y, Fukagawa K, Kinoshita S (2008). Prevalence of dry eye disease among Japanese visual display terminal users. Ophthalmology.

[27] Zhu W, Xue L (2018). Systemic Comorbidities in Dry Eye Patients: A Case-Control Study. Journal of Ophthalmology.

[28] Cobanoglu RK, Vega J, Burgos C, Erkan D (2025). Assessment of triple antiphospholipid antibody-positive patients based on clinical and laboratory domains of 2023 ACR/EULAR antiphospholipid syndrome classification criteria. Semin Arthritis Rheum.

[29] Ismail MF, Khalafalla I, Qarbote AI (2025). Prevalence of dry eye disease in African populations: a systematic review and meta-analysis. BMC Ophthalmol.

[30] Ozek D, Kemer OE, Omma A (2019). Association between systemic activity index and dry eye severity in patients with primary Sjögren syndrome. Arq Bras Oftalmol.

